# Philadelphia County Medical Society—Disputed Points in Hysterectomy1Read April 12, 1893.

**Published:** 1893-08

**Authors:** Joseph Price

**Affiliations:** Philadelphia


### PHILADELPHIA COUNTY MEDICAL SOCIETY.—DISPUTED POINTS IN HYSTERECTOMY.[1]

BY JOSEPH PRICE, M. D., Philadelphia.

THE mooted questions in surgery grow less as our experiences enlarge and ripen. There is in our science and art some certainties, some points upon which there is unanimity of enlightened opinion. There is however, also, as in all other sciences and arts, as in all other lines of human enterprise and endeavor, disputed points; disputed, we must take it, from the standpoint of conscientious opinion. These differences are the chief factors, the motor forces of our advances. Without them inertia would take the place of our activities. The fact of our advances is not disputed; the lines along which they have been made, direct the way of interesting and instructive study. We have a profound interest in the names and work of those toiling pioneers who have blazed the trees for our guidance to lessen the difficulties of our following. What they have done for womankind will always lie beyond the power of biographical pen to narrate. We would find it difficult to distribute our debt of obligation when we come to consider the great labors, the brilliant work of McDowell, Kimball, and the Atlees, of Péan, Keith, Koeberle, Hegar, Billroth, Kaltenbach, Kleeberg, Schroeder, Lawson Tait, Bantock, Thornton, and others. We find stimulus in such names and such records for worthy following. They have given us the sublime lessons of their experience. What masters they are—all of them! They represent the genius of science, of practical skill; they have enlarged our resources; they have helped us to make many lives worth living. Some of these men are living today, are yet giants at the wheels, yet students in the solution of great surgical problems.

In considering the definitions of hysterectomy we must bear in mind nomenclature. Schroeder's term, myomotomy, is not synonymous with hysterectomy; is not hysterectomy; it more appropriately applies to simple extirpation of the tumor. Hysterectomy is the removal (Kimball's operation) of the whole body, or any section of the uterus, with tumor inseparable therefrom. Such high authority as Thornton places within its field all cases in which the uterine cavity is laid open and more or less of its wall removed along with the fibroid; whether one or both ovaries is also removed, is a matter of no consequence. Sometimes it is

1. Read April 12, 1893.

more convenient to remove one or both, applying the term vaginal hysterectomy to cases in which fibroids, the uterus, and the uterine appendages are all removed.

The progress made in perfecting the operation has taken some disputed points out of the field. Experience has given something of definiteness to our views; still there are two camps. The disputed points involve methods, rather than questions, of the justifiability or safety of the operation; on these points there is unanimity of sentiment among experienced surgeons. There may be yet some division of opinion as to what cases should be operated on, and what cases should be let alone. The operation was long regarded as one of the most fatal in surgery. The low rate to which the mortality following the operation has been reduced where the cases fall into experienced and skilful hands, has given it an abiding and important place among the life-saving procedures. In the matter of methods, men are likely to credit those methods with being best which, by their own tests and in their own individual and professional experience, have given the best results. One or more failures with any one particular method of procedure drives some men to try others. With their first success they christen the baby, "My method," "My modification," "My improvement," or "My invention," and the entire profession is exceedingly glad that a new genius has been born into the profession—that there is a new light in Israel.

The history of the treatment of the pedicle in ovariotomy has influenced all of the older ovariotomists to try the same methods and materials to perfect an intra-peritoneal method in hysterectomy. The early efforts of Schroeder were quite successful. Some of the younger operators have improved the statistics by clean extirpation, but we yet remain in two camps as to the management of the pedicle.

Operators clinging to the nœud and the extra-peritoneal method are making the best showing, operating right along with a very low mortality. It cannot be inferred from the success of the intra-peritoneal method in ovariotomy that improved or equally successful results will be attainable by the intra-peritoneal method in supra-vaginal hysterectomy. The results in many large and ripe experiences establish the fallacy of this idea; such inference is in blind disregard of essentially different conditions. Ligatures cannot be safely used in uterine fibroid, or myomatous tissue. Silk, as applied to the pedicle in cystomas, is harmless and safe.

I would say here that the earlier errors in diagnosis, mistaking cystiform degeneration, fibroids, or edematous myomas, for ovarian cystoma, were common, and the cases were either abandoned or incomplete operations done with disastrous results. Some of the most skilful operators did not escape making these errors.

The treatment of the pedicle has been repeatedly and exhaustively discussed. Results have dampened the enthusiasm of the advocates of the intra-peritoneal method.

It is necessary in the removal of about all fibroids to make a pedicle. Its manufacture in extra-peritoneal hysterectomy is the one important feature of the operation. It should be made small. Suturing securely against hemorrhage is also the important feature in the intra-peritoneal, and the avoidance of hemorrhage and the ureters are the important features in the extirpation method.

Shock is minimized in the extra-peritoneal method, the operation being shorter, exposure and manipulation less, than in any of the intra-peritoneal methods.

The methods of turning the pedicle into the vagina is a tedious operation ; the risks of hemorrhage and of injury to the ureters is even greater than that of a clean extirpation of the cervix.

The question is often asked, Why leave the cervix or stump in at all ; it is the most common source of hemorrhage and sepsis in all the intra-peritoneal methods ? Its removal is the perfected operation, but the results as yet have not been as good as in the extra-peritoneal method of treating the stump.

Hemorrhage is incident to the supra-vaginal, as it is to all the methods. The bleeding varies greatly, and sometimes is absent altogether. In this procedure the elastic ligature (Kleeberg's) and the wire ligature minimize the risks of hemorrhage. The chief danger in the intra-peritoneal method is bleeding from the pedicle. Drainage, or the dry treatment, where adhesions have been extensive, is of vital importance in these operations. It is an important object to get and keep the stump dry. In some cases the dressings need not be changed for a week or more. They should be changed when they become moist. The advantage should be kept in mind of sewing the edges of the peritoneum across the stump, thus preventing retraction when the loop has become somewhat loose from the shrinkage of tissue. The duration of the operation is one of the many factors to be considered. There should be that rapidity consistent with due caution and scrupulous attention to essentials. There is no time for fussiness. There is the shock of the anesthetic. Extensive adhesions, bowel and bladder complications, require painstaking surgery, and tedious and slow the steps of the procedure, and somewhat lengthy, however deft and educated the hands engaged. Temperature is an important consideration. Supplying dry heat throughout the operation will avoid, to a very great extent, the shock due to the chill of the atmosphere. In the matter of shock, long exposure and long anesthesia count for much. It should be kept in mind, however, that to deal with an abdominal wound carelessly or too hurriedly is bad surgery. Every step should be timed to the needs of the case, every motion those of a master-workman, and there should be summoned into service every resource of our science and art.

When we come to consider hysterectomy in all its phases, the condition of the patients when they come into our hands, the dire extremity that drives them to us, that they come to us with general health broken down, often complete physical wrecks, and familiar as we are with resultant issues—we have no difficulty in appreciating the difficulties we have to encounter. The professional responsibility is a heavy one. The patient's condition suggests the urgent question: What should be done?

We appreciate the truth of J. Knowsley Thornton's statements; we accept them in the main as surgical truths, into the acceptance and practice of which the profession should be educated. As to the relative value of two very different surgical procedures for the cure of fibroid enlargements of the uterus, he says:

> I feel that I am confronting one of the most difficult questions in abdominal surgery armed with imperfect weapons. Medicine has long and vainly endeavored to deal satisfactorily with this disease, and now the surgeon's aid is invoked. I do not deny that many cases have been relieved by medical treatment, and that some have been cured while under such treatment. I do think, however, that it is an open question how many of the cases cured while under treatment were cured by the treatment, and I believe the majority of such cures have been due to the coincident interposition of Dame Nature.
>
> A very large number of patients never suffer pain, or even inconvenience, enough to make them consult either physician or surgeon. But admitting all this, there undoubtedly remain a large number of cases urgently demanding surgical aid. Some patients are brought face to face with death from hemorrhage, excessive growth of the morbid elements, or constant interference with rest from pain and discomfort. Others are gradually but surely reduced in strength, and have lesions of vital organs as the result of constant pressure and displacement. When surgical treatment is spoken of, we are told that we have no right to interfere with fibroids as we do with ovarian tumors, because the latter surely kill if left alone, and the former do not. I am certain that this argument is only partly true, and everyone who sees a large number of cases will bear me out in the statement that numbers of women die every year from the direct and indirect effects of fibroid enlargements of the uterus.

I would ask, How much of the general surgery of the day which is dangerous to life would continue if surgeons ceased to perform operations of expediency, that is, to operate for deformities and diseases which do not endanger life in themselves, though they deprive their victims of all the pleasures of life ? I affirm, then, that there are many cases of fibroid enlargement of the uterus which endangers the lives of their bearers, and that there are many more which make these poor suffering women so miserable and useless that they are justified in running the risks of operation, and that the surgeon is justified in operating. We must remember that these operations are usually undertaken in extreme cases, and when the patients are worn out with disease and suffering.

The operation of complete supra-vaginal hysterectomy, with removal of both ovaries, has become, when properly performed, one of the most successful of the great operations.

Hegar and Kaltenbach, by their new extra-peritoneal method, have saved eleven cases out of twelve, and the surgeons at the Samaritan Hospital have in the last year had equally successful results, also by the extra-peritoneal method, using Koeberle's wire serre-nœud in much the same way that Hegar used the elastic ligature. These operations of hysterectomy and complete supra-vaginal hysterectomy still remain, however, very formidable operations. They are terrible mutilations ; the patients are slow in convalescence. Is there, then, no operation of less danger, of quicker convalescence, and of better and more perfect results which we, as surgeons, can recommend to our patients.

Thanks to American surgery, the brilliant conception of Blundell, in 1823, was made a recognized surgical procedure by Batty in 1874, and from the labors of Hegar, Trenholm, Tait, Savage, and others, I am able to present to you a perfected operation, which will render this formidable hysterectomy still less often necessary in the future than it has been in the past.

The complete removal of the uterine appendages, when efficiently performed, cures fibroids of the uterus with rapidity and certainty, And I will ask you to remember that this operation is not such a serious mutilation, and does not leave behind it any mark except a small linear scar on the perfectly closed abdominal parietes. The removal of the uterine appendages is attended with infinitely less danger to life than are the various operations for the removal of uterine fibroids.

Are we, then, justified in subjecting our patients to the formidable operation of supra-vaginal hysterectomy when we can cure them by removal of the uterine appendages?

It should be accepted as a settled fact that we are never justified in doing a hysterectomy when the appendages can be removed early in the growth of the tumor.

DISCUSSION.

Dr. Charles P. Noble: It has been a very short time since we were all firmly imbued with the idea that for fibroid tumors, practically, we should never operate. They were considered to be benign tumors, not endangering life, and it was held that by the use of ergot, muriate of ammonia, etc., the serious symptoms could be combated. It, however, did not take a long experience to convince me that fibroid tumors are much more serious than our elders taught, and I am quite certain that it is the experience of every one that fibroid tumors do bring patients to the brink of the grave, and even cause death, either by long exhausting hemorrhages, or by attacks of peritonitis, (although such attacks are usually due to coincident disease of the tubes,) or by pressure on other organs in the pelvis, especially the ureters. In addition, when fibroids attain any size, they may degenerate into fibro-cysts, or be converted into sarcomata. There is no doubt that not an inconsiderable number of fibroids become malignant. A consideration of these facts has determined me that hereafter I shall operate on fibroid tumors which cause much hemorrhage, or much suffering, or have attained considerable size.

I agree with Dr. Price, that when the fibroids are small, the removal of the appendages is a simple, safe, and curative operation. In all the cases in which I have done this operation, the results have been all that could have been expected. In every case the patient recovered, and in every case the fibroids became much smaller, the hemorrhages ceased, and the patients were symptomatically cured. I think that an exception should be made where there is a single fibroid which can be removed from the uterus, and the bed from which the fibroid has been removed can be closed by stitches, thus leaving normal appendages and practically a normal uterus. I have never done this operation, but I have had several patients under my care who had been operated on in that way, and the results were all that could be desired.

With reference to hysterectomy, I believe that the technique of this operation is undergoing a change; and while at the present time the extra-peritoneal method with the use of the serre-nœud may be giving the best results, I have no doubt that in the near future two other methods of doing hysterectomy will give equally good results, and they have certain advantages over the serre-nœud, and will, I think, come to supplant that method. I refer, first, to the method of tying off the broad ligaments down to the vagina, separating the bladder in front and amputating the cervix and stitching the peritoneum over the cervical stump. This operation has been done most frequently in this city by Dr. Baer, and his results have been most excellent. I have done the operation. It is a simple operation, and I believe that it is destined to supplant the use of the serre-nœud. The second method to which I have referred is the complete extirpation of the uterus. With the patient in the Trendelenburg posture it is a simple matter to remove the entire uterus, and when the patient is in good condition it can be done without any marked increase in the length of time which the operation takes. If it is desired, the ligatures can be brought out into the vagina. We then have a perfectly clean peritoneum with a simple seam extending from one side to the other. It is not necessary to make use of drainage from the abdominal wound.

Both of these methods have advantages over the use of the serre-nœud. In them you have no stump to slough. Both are extra-peritoneal just as much as the method with the serre-neud. In the two methods the convalesence is shorter and there is less danger of hernia. For these reasons I have no doubt that complete extirpation or amputation at the level of the vagina will supersede the method with the serre-nœud.

Dr. J. M. Baldy: I shall have nothing to say in regard to removal of the appendages, for I think that we all agree that small fibroids can be successfully treated by this method.

I have, however, considerable to say in regard to hysterectomy. I think, as the essayist tonight has said, one is apt to use that method, and to consider it the best, which in his experience has given him the best results. I was sorry to hear Dr. Noble speak of extirpation of the uterus as extremely easy. Total extirpation of the uterus is the hardest operation in the whole range of surgery. I have come across nothing that has equaled the difficulties and the complications to be met with in the complete removal of the uterus from above. I do not believe that it will take the place of the extra-peritoneal method of dropping the stump and leaving the pedicle.

The extra-peritoneal method with the serre-nœud is the only method the beginner should think of using; but those who are skilled and are familiar with the anatomy, both normal and distorted, will not rest satisfied in the failure with treating the stump extra-peritoneally. The old objection to treating the stump intra-peritoneally was hemorrhage from loosening of the ligature, which was applied to uterine tissue. At present, no one applies the ligature to uterine tissue. The ligatures are placed in the broad ligament tissue as in the vaginal hysterectomy, and there is no danger from hemorrhage if the bleeding is controlled before the wound is closed. There is no more danger of sepsis than in the extra-peritoneal method, for if the operation is properly completed the stump will really be extra-peritoneal. The after-suffering of the patient is lessened to a great extent. The distortion of the ureter and of the bladder is done away with entirely. With the extra-peritoneal method it is necessary to keep the patient in bed six weeks to two months, in order to guard against hernia. By dropping the stump, the patient is out of bed as soon as after an ovariotomy, and there is no more danger of septic poisoning, fistula, or hernia, than after any exploratory operation. There is, however, more danger of shock where the pedicle is dropped, for the operation is a longer one. If the condition of the patient will not warrant keeping her half an hour longer on the table, dropping of the stump should not be considered, but the stump should be treated extra-peritoneally. In regard to danger to the ureters, I have only once seen the ureter in fifty cases, and have never tied it.

I would not agree with the statement that the best showing is made by the extra-peritoneal method. I think that the time has come when the statistics of the intra-peritoneal method fully equal those of the extra-peritoneal. I have reported some twenty-seven cases in which I treated the stump extra-peritoneally, and about ten in which I dropped the stump, and my results have been equally good.

Dr. B. F. Baer: I agree with much that Dr. Price has said, and especially as to the advisability of early operation in fibroid tumor. In many instances these tumors continue to grow, and sometimes undergo malignant change after the menopausal age is reached.

My recent experience only confirms the opinion which I have for several years held upon this subject. The majority of cases upon which I have operated had either reached the menopause or had passed it. The last case operated upon, last Monday, was a patient forty-eight years of age, who had a large fibroid tumor which had been growing for ten or twelve years, and which had given rise to the ordinary symptoms of that disease, as hemorrhage, pressure, etc. She was advised to wait until the menopause, but when she reached that age the tumor increased rapidly in size, especially so during the last year. The operation showed it to be an edematous fibroid with a malignant appearance, I fear is sarcomatous, although a microscopic examination has not yet been made.

Three months ago I performed a hysterectomy upon a lady fifty-five years of age, for a growing fibroid tumor. The specimen showed multiple fibroid degeneration of the uterus, one of which was breaking down and was undergoing malignant change. This and similar cases, a number of which I have had during the last two years, convince me that we cannot too soon get rid of the idea that the menopause cures these cases. I believe that where it is determined that a fibroid tumor of any size exists, the patient is safer if the uterus is removed. The teaching that fibroid tumor is a benign disease, that it never destroys life, and that if the patient reaches the menopause she is safe, is erroneous. Even where no symptoms are present, such as hemorrhage or pain, the constant presence of a tumor induces such an unhappy mental state, that, where the patient desires it, the tumor should be removed.

My experience with oöphorectomy in cases of fibroid tumor has not been as encouraging as that of some of the speakers who have preceded me this evening. Even if the ovaries and tubes have been entirely removed, the tumors do not undergo atrophy, or do it in such a slow manner that the patient is dissatisfied with the result. Hemorrhage often continues, and pain and pressure are not relieved.

Eighteen months ago I removed the diseased ovaries and tubes from a patient who had a number of small fibroid tumors of the uterus. The whole mass did not extend much above the superior straight. I had hoped that the removal of the appendages would cure the patient, and she was much improved for about six months. She then began to bleed, and the hemorrhages recurred so frequently and were so profuse that she again sent to me. Examination at this time showed that the fibroids were growing. Hysterectomy was then done, and she is well with the result. I believe that hysterectomy by the method which I advocate, and in experienced hands, is as safe, if not safer, than ovariotomy, and I have no doubt that the patient is better, because she has gotten rid of the tumor at once.

I was surprised at the position taken by Dr. Price this evening. I had hoped that he would be willing to make what I regard as an advance, and would report to us that he had at least tried this new operation in one case, because I am firmly of the belief that it is the most scientific method. I am not alone in that belief, for as we have heard just now, other eminent operators are taking it up. I have no doubt but that it will be regarded as the only practical and safe method as soon as it is fully understood. This operation leaves the cervix extra-peritoneal, as Dr. Noble has said, even more so than by fixing it in the lower angle of the wound. It leaves it in its subperitoneal or extra-peritoneal position just as the uterus is anatomically. The incision is closed without drainage, the parts left to heal primarily and without interference, and they do so in every instance. The convalescence is shortened a month, at least; there is no distortion of bladder and bowel, and no after-dragging upon the cicatrix.

Of course, this operation requires experience and skill, and should not be undertaken by the beginner in abdominal surgery. The way for a beginner is not to begin with hysterectomy. He should first serve an apprenticeship in the more minor operations in this field. But this will apply to hysterectomy by any method, for it should always be regarded as a very major operation.

Dr. Thomas S. K. Morton: I wish to say one or two words about this intra-peritoneal operation. I had the pleasure of seeing Dr. Baer perform it recently, and it was more or less of a revelation to me. The operation was done by ligating the arteries of the broad ligament on each side. The uterus and appendages were then separated from the broad ligaments, and the cervix was cut, leaving one-half or three-fourths of an inch of uterine tissue. The broad ligaments then slipped down around the cervix, making a straight line, which required no sutures. The operation did not seem much more serious than an ovariotomy, although the incision was longer and more surface exposed.

Dr. Charles P. Noble: In regard to injury of the uterus, I think that the danger is less by the method referred to by Dr.

Baer than by the nœud. By tying close to the uterus, there is less risk of tying the ureters. In doing complete extirpation by the method advocated by Dr. Polk, of New York, I think that there is considerable danger of including the ureter in the ligature around the uterine artery. In regard to the confusion of the terms extra- and intra-peritoneal, I wish to emphasize the fact that in both of the methods the stump is extra- and not intra-peritoneal. It is extra-peritoneal, as the stump is under the peritoneum, and the peritoneum is sewed over the stump. In the one case the remnant of cervix, in the other the broad ligaments, constitute the stump. It is as much extra-peritoneal as though the peritoneum of the abdominal parieties were stitched around it on the anterior abdominal wall. It is, therefore, an extra-peritoneal method of treating the stump.

Dr. Price: I look upon hysterectomy as one of the most serious operations. I have had a series of 103 cases, and lost six cases in the first hundred, and one in the last three. Some were simple hysterectomies, but ninety per cent. were very complicated. I have known more women to die from neglect than from operation. In the past the intra-peritoneal methods have been much less satisfactory than the extra-peritoneal. The so-called intra-peritoneal or drop methods have unquestionably been improved, but the very best results have been attained by some German operator by clean and complete extirpation. Hegar has had a series of twenty-two cases without a death. The supra-vaginal, extra-peritoneal method is much the simpler one, and if you remove a healthy tumor from a healthy peritoneal cavity, and make the pedicle at the internal os, there is no reason why the case should not get well. It is only the complicated cases that you lose.

Suppurative forms of tubal and ovarian disease must bear a causal relation to fibroid tumors. I rarely remove a fibroid without finding an occluded tube or suppurative form of disease.

A good deal has been said about complete extirpation. If you ligate and place two or three pedicles in the vagina and match the peritoneum above, you have an extra-peritoneal method. If you apply forceps, you can match the peritoneum above and have the pedicle in the vagina.

The merits of a method should not be judged by a few isolated triumphs. In the hands of a few, the intra-peritoneal method of dealing with the stump has been measurably successful, yet the statistics of results, as well as the weight of surgical opinion, strongly favor the extra-peritoneal method. The greater uniformity of successful results is with this procedure. Through it we can deal better with the visible and the obscure complications. One of the factors of our better success in hysterectomy is that we have less ignorant meddling with the stump of the pedicle. In these operations, as in others, there should be no morbid products left behind, nothing that can produce recurrence of disease. There doubtless will be further improvements in the mechanical appliances employed in our procedures, but there is something more in the work than the merely mechanical part. Too many instruments discredit our skill.

